# Individual preferences for task coordination strategies in multitasking: exploring the link between preferred modes of processing and strategies of response organization

**DOI:** 10.1007/s00426-020-01291-7

**Published:** 2020-01-31

**Authors:** Jovita Brüning, Jessika Reissland, Dietrich Manzey

**Affiliations:** Work, Engineering and Organizational Psychology, Technische Universitaet Berlin, Marchstrasse 12, F7, 10587 Berlin, Germany

## Abstract

Recent investigation of individual differences in multitasking revealed evidence for individual preferences for modes of task processing (serial vs. overlapping) in a task switching with preview (TSWP) paradigm and different strategies of response organization (blocking, switching, and response grouping) in a free concurrent dual-tasking (FCDT) paradigm. However, this research on individual differences at the levels of cognitive task processing and behavioral response organization has been pursued separately, thus far, by testing independent samples of participants. In the current study, we investigated whether these two levels of task coordination were linked intra-individually. As individuals preferring an overlapping task processing mode can generate time gains particularly at task switches, we predicted that they prefer a switching strategy of response organization. In contrast, individuals preferring a serial processing mode are expected to prefer a blocking strategy to reduce dual-task demands. These predictions were confirmed in an experiment based on *n* = 70 participants. Indeed, most serial processors preferred a blocking strategy, whereas overlapping processors predominantly preferred to switch between the tasks. This finding suggests a strong correspondence between individual preferences emerging in both aspects of task coordination, which might reflect a common basic difference in the preferred style of cognitive control (flexible vs. persistent). Moreover, in case the preferred modes of task processing and strategies of response organization did not correspond to each other, the overall multitasking efficiency was comparably low. Thus, the distinction between the preferences for both aspects of multitasking seems to be an important aspect of understanding multitasking performance and should be considered in future studies.

## Introduction

In our daily lives, we are often confronted with the demand to multitask—that is, to cope with two or more tasks overlapping in time. To this end, more than one task-set must be maintained concurrently, and ways need to be found to coordinate the concurrent tasks effectively (Koch, Poljac, Müller, & Kiesel, 2018). Our basic assumption of the present study is that elementary challenges of task coordination in multitasking involve two interwoven aspects, which we refer to as task processing and response organization.

Task processing concerns how the internal processing of multiple tasks is organized. It involves, for example, issues such as serial versus overlapping processing, or maintaining and activating different task-sets. For the last 40 years, this aspect of multitasking has been the main focus of interest, involving two different lines of research. These two lines include research on central limitations of parallel task processing based on the psychological-refractory-period (PRP) paradigm (Pashler, 1994a; Telford, 1931), and research on cost effects when switching between tasks based on different variants of the task-switching paradigm (Kiesel et al., 2010). Both paradigms focus on a high control of timing of tasks and a prescribed sequence of tasks, resulting in no or only very few degrees of freedom for own response organization, which makes them particularly suitable to reveal specific constraints involved in task processing. As a result, single mechanisms could be identified, including but not limited to central limitations in response selection (Pashler, 1994a), issues of crosstalk and stimulus–response bindings (Hazeltine, Ruthruff, & Remington, 2006; Hommel, 1998; Navon & Miller, 1987), or cost effects due to a reconfiguration of task-sets when switching between tasks compared to task repetitions (Monsell, 2003). These mechanisms represent constraints of cognitive processing humans have to cope with when performing multiple tasks concurrently.

In contrast, issues of response organization particularly arise in completely self-organized multitasking and involve the aspect of how responses to concurrent tasks are organized over time, reflected in a certain sequence of behavioral responses to the different tasks. Thus, response organization can differ in respect to when and for how long a person works on each of the concurrent tasks. The standard paradigm, reflecting such demands, is represented by the typical concurrent dual-tasking paradigms of the 1970s and 80s (Navon & Gopher, 1979). Here, participants are required to work concurrently on two independent streams of tasks (Wickens, Mountford, & Schreiner, 1981). Usually, they are instructed to maximize their performance by achieving as many trials of both tasks as they can within a given time window, without further instructions or constraints on how to achieve this. That is, participants have full control over how often they repeat one task, switch between tasks, or whether or not they group their responses. Studies using this paradigm have usually addressed performance only on a more holistic level, using relatively global performance measures of multitasking efficiency and task interference (Navon & Gopher, 1979; Wickens, 2002). Thus, they have acquired broad knowledge about which tasks can or cannot be performed concurrently with considerable efficiency, but largely neglected any detailed investigation of the strategies of response organization involved in this performance (see for an exception, Damos & Wickens, 1980). Other paradigms involving issues of response organization in addition to task processing are variants of task-switching or PRP paradigms providing at least some degrees of freedom for individual response organization. Examples are the voluntary task-switching paradigm (Arrington & Logan, 2005), or PRP paradigms, which do not impose a specific response sequence by means of stimulus presentation or instruction, thus providing degrees of freedom for individual choices of how responses are organized (De Jong, 1995; Kübler, Reimer, Strobach, & Schubert, 2018; Pashler, 1994b).

Interestingly, it seems that individuals differ in their preferences of how they process tasks and how they organize responses during multitasking. In this context, preferences can be understood as a stable tendency (comparable to a bias or affinity) how individuals spontaneously deal with multitasking demands. The existence of such preferences is suggested by a variety of incidental observations and anecdotes, which can be found in the literature (e.g., Damos & Wickens, 1980; Jersild, 1927; Kessler, Shencar, & Meiran, 2009; Schumacher et al., 2001). For example, already in the very first experimental series on task switching, Jersild (1927) observed that a subgroup of participants did not show switch costs but switch benefits. Based on this observation, he assumed that these participants had overlapped the processing of both tasks, whereas others tended to process the tasks in a strict serial manner. Similarly, observations of individual preferences can be found in results of PRP research that investigates the effects of different practice schedules on the PRP effect (i.e., the prolongation of the response to the second task after a short stimulus onset asynchrony). The results suggest that even after extensive practice of one of the component tasks or both tasks, not all participants were able to eventually reduce the PRP effect to a negligible minimum (Maquestiaux, Lague-Beauvais, Ruthruff, & Bherer, 2008; Maquestiaux, Ruthruff, Defer, & Ibrahime, 2018; Schumacher et al., 2001, Exp. 3).

Individual differences in the way responses are organized become particularly apparent when experimental paradigms are used that offer at least some degrees of freedom in this respect. An example from PRP research includes the common finding that, if no definite sequence of responses is required, participants do not always organize their responses to the tasks in a strictly serially manner but tend to group their responses together in a considerable number of trials (Ulrich & Miller, 2008). Other observations include individual differences in the so-called repetition bias in voluntary task switching (Kessler et al., 2009; Mittelstädt, Dignath, Schmidt-Ott, & Kiesel, 2018; Mittelstädt, Miller, & Kiesel, 2018), or the observation of different strategies of response scheduling in concurrent task performance, for example, massed, alternating, or simultaneous responding (Damos & Wickens, 1980).

To explore both aspects of individual differences in multitasking in a more systematic manner, two different paradigms have been proposed (Reissland & Manzey, 2016), which also were used in the present study. The first one is the task-switching with preview (TSWP) paradigm, which enables to identify individual preferences with respect to a serial versus overlapping mode of task processing (Reissland & Manzey, 2016). In classical task switching paradigms, only the stimulus of one of two tasks is visible at a time, thus, requesting a strict serial processing of both tasks (Kiesel et al., 2010). In the TSWP paradigm, the stimuli of both tasks are always visible concurrently, with a cue marking the task that has to be performed in the current trial (i.e., the currently relevant task). After each response to the relevant task, only the stimulus of this latter task is updated, while the stimulus of the second task remains. Thus, while working on the marked (relevant) task, the concurrently visible task stimulus of the other task serves as a preview to the stimulus, one has to respond to after the next task switch (Spector & Biederman, 1976). By presenting the task stimuli in this manner, combined with a regular task sequence (e.g., AAABBB), the TSWP principally allows but does not enforce an overlapping processing of the switch stimulus while still performing the currently relevant task. Thus, it provides degrees of freedom for different modes of task processing, but still imposes a certain pattern of response organization (e.g., three times responses to task A followed by three times responses to task B). Using this paradigm, Reissland and Manzey (2016, Experiment 1) found two subgroups of participants differing in whether they made use of the preview to optimize task switches. Thereby, they substantiated previous anecdotal observations of individual biases concerning a more serial versus overlapping mode of task processing (Jersild, 1927) by a more systematic approach. A study by Brüning and Manzey (2018) confirmed these results based on a larger sample size (*n* = 45), but also found a third subgroup of participants that could not be unambiguously classified as serial or overlapping processors (i.e., so-called semi-overlapping processors). Besides, they showed that at least the individuals using an overlapping processing mode were able to flexibly change to a more serial mode of processing in case of increased risk of crosstalk between tasks. This suggests that the spontaneous preference for a certain mode of processing is not a rigid predisposition, but a kind of cognitive style that can be flexibly adapted to the current environmental constraints and might be at least partly under voluntary control.

**Fig. 1:**
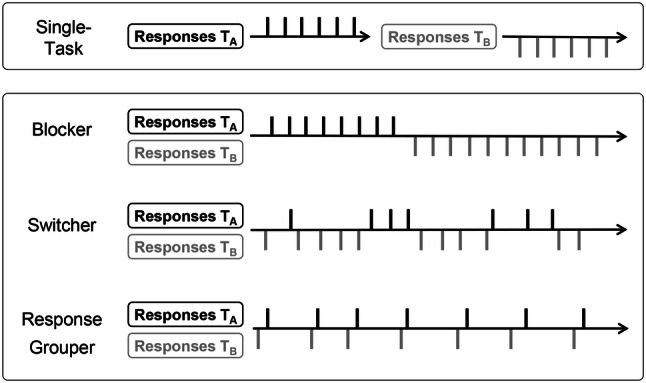
Stereotypical sequences of responses to both tasks, reflecting the three different strategies of response organization typically observed in the free concurrent dual-tasking (FCDT) paradigm

To additionally explore individual preferences at the level of response organization strategies, Reissland and Manzey (2016) used what we refer to as free concurrent dual-tasking (FCDT) paradigm. This paradigm directly corresponds to the concurrent dual-tasking paradigms from the 1970s and 80s as described above. Similar as in the TSWP, the stimuli of two streams of tasks are always concurrently visible. However, beyond that, participants in the FCDT are also completely free to decide when and for how long they want to work on a given task. Using this paradigm, combined with applying a detailed post hoc analysis of individual response patterns, Reissland and Manzey (2016) could identify three different strategies of response organization. One subgroup of individuals used a blocking strategy; that is, they focused on one task for a considerable number of trials (i.e., 65 repetitions on average) before switching to the other. Another subgroup of individuals preferred a switching strategy, reflected by switches between tasks about every 4–7 trials. The remaining individuals permanently alternated between both tasks. However, a closer look at the time characteristics of their response patterns showed a remarkable similarity to a response grouping pattern described previously in the studies by Damos and Wickens (1980) and Damos, Smist, and Bittner (1983). Stereotype response patterns reflecting the different strategies are shown in Fig. 1. The blocking strategy seems to be motivated by keeping concurrent tasks as separate as possible, thereby reducing multitasking demands to a possible minimum (i.e., only maintaining two task-sets). In contrast, the other two strategies have in common that individuals strive for some sort of task interleaving as a means of coordinating multiple tasks.

The replicable findings of individual preferences for different modes of task processing in the TSWP paradigm and certain strategies of response organization in the FCDT paradigm raise the question of a possible link between these two aspects. Although it seems plausible that both aspects of multitasking are interwoven, it is still unclear to what extent the way in which an individual cognitively prefers to deal with multitasking requirements directly translates into the temporal organization of responses to the tasks. At least the findings of different styles of response organization in voluntary task-switching and in PRP research with SOA = 0 referred to above suggest that modes of task processing and behavioral response organization do not just represent two sides of the same coin. Instead, they are two aspects of multitasking, which, at least to some extent, could be combined in different ways. For example, in the PRP paradigm, the same serial processing at the stage of response selection can be associated with different sorts of response organization (e.g., serial responding or response grouping, see Pashler, 1994b).

The present study particularly aimed to examine possible links between the two aspects and how they both interact in determining the overall multitasking efficiency. Specifically, we assumed that the way how individuals prefer to process the tasks in the TSWP (serially vs. overlapping) ideally would be predictive for how they organize their responses when performing the FCDT to find the best compromise between maximizing performance while at the same time minimizing possible costs of multitasking.

For example, if individuals strongly prefer a serial task processing mode in the TSWP paradigm, thus striving to shield the processing of the currently relevant task from the processing of the preview stimulus, the frequent imposed switches in this paradigm lead to severe performance decrements by accumulating switch costs (Brüning & Manzey, 2018; Reissland & Manzey, 2016). However, with the possibility for free response organization in the FCDT, serial processors can avoid these costs by choosing a strategy of minimizing switches, that is, by working on longer sequences of one task before switching to the other. Thus, for individuals preferring serial processing in the TSWP paradigm, we expected a corresponding preference for the blocking strategy in the FCDT paradigm.

In contrast, overlapping processing during the fixed sequence of the TSWP paradigm allows benefiting from the stimulus preview by generating considerable time gains at switch trials. Hence, individuals with a preference for overlapping processing should strive to switch between tasks to exploit options to save time. As the FCDT paradigm provides both the opportunity to freely schedule task switches and to preview the task stimulus which they would switch to, it should be especially appealing to overlapping processors identified in the TSWP paradigm to organize their responses in the FCDT paradigm in a way that supports overlapping processing. Thus, we predicted that individuals preferring an overlapping processing mode in the TSWP paradigm preferred interleaving strategies of response organization (i.e., either switching or response grouping) in the FCDT paradigm.

Regarding the semi-overlapping processors, the expectations for according links to response organization strategies were less clear-cut, though. Since they do not exhibit a distinct preference for either mode of processing, both could be feasible for them. Hence, the according links for semi-overlapping processors were investigated in an exploratory manner.

To examine the proposed link between both aspects of task coordination in multitasking, we investigated the performance of the same participants in the TSWP and FCDT paradigm. The same set of simple classification tasks as in the study by Reissland and Manzey (2016) were used. Links between the emerging individual differences on both levels of task coordination probed by the different paradigms were examined.

In addition, we were interested in how the resulting combinations of both task coordination levels affected the overall multitasking efficiency in terms of task throughput in blocks where the two tasks had to be performed according to a prescribed schedule (TSWP) or in a self-organized manner (FCDT), relative to single-task performance. Based on the assumed link between modes of processing and response organization strategies, we expected that individuals preferring the overlapping mode of task processing in TSWP and a corresponding interleaving strategy in FCDT would achieve a higher overall dual-task efficiency, reflected in multitasking benefits than participants preferring serial processing and a blocking strategy. The former group should benefit from the preview during switching, whereas the cautious strategy of the latter group should lead to neither costs nor benefits. Worst overall multitasking efficiency in terms of task throughput (how many of the two tasks can be accurately performed in a given time) was predicted for participants preferring serial processing but applying one of the interleaving strategies of response organization in self-organized multitasking, that is, strategies which would accumulate performance costs due to repeated task switching.

## Methods

### Participants

Seventy-three volunteers participated in the study. The data set of one participant had to be excluded due to high error rates (ERs; > 15%) in both tasks. Two further participants were excluded, because they showed no coherent response strategy. They blocked their responses for about 70% of the time, but started to group their responses in the middle of the experiment. For the remaining 70 participants (46 females), a clear preference could be found for both levels of task coordination. Their ages ranged from 18 to 33 years (mean age = 25.5 years, standard deviation, SD 3.5 years); they had normal or corrected-to-normal vision, were either right-handed or ambidextrous, and were able to speak German at native language level. All participants received 11.25 Euro or course credit, and an additional monetary bonus for each correctly answered stimulus, which could accumulate up to five Euro.

### Paradigms

#### Task-switching with preview

**Fig. 2:**
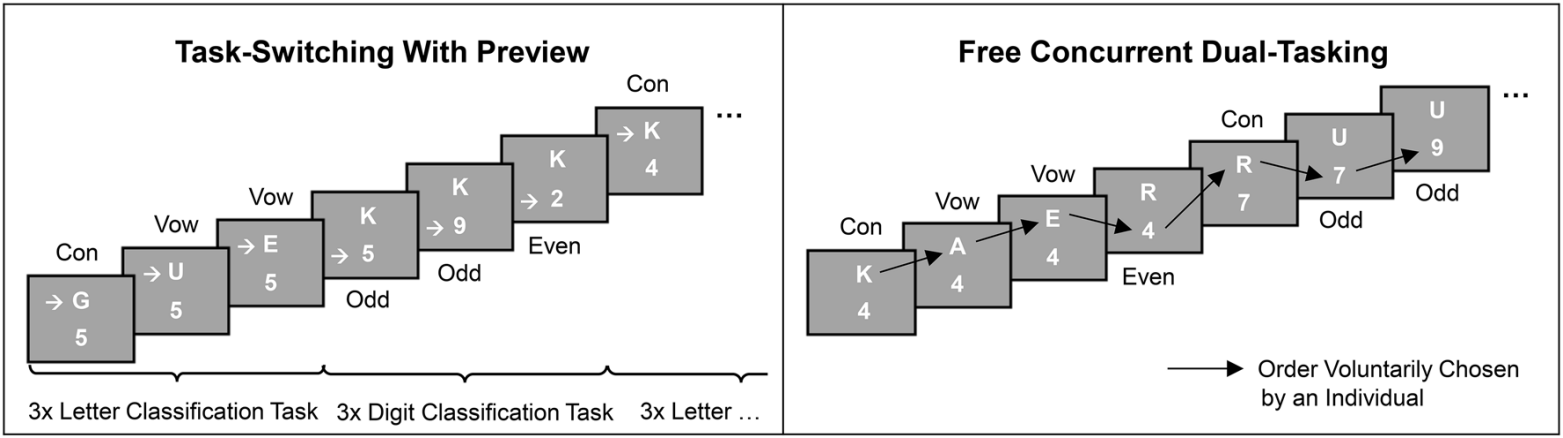
Trial sequences in the task switching with preview (TSWP) paradigm, and in the free concurrent dual-tasking (FCDT) paradigm. The scheme includes an exemplary stimulus presentation in dual-task blocks with the tasks requiring the classification of digits (odd vs. even) and letters (vowel vs. consonant). Upon each given response, a new stimulus was displayed directly for the task being performed (response–stimulus interval = 0 ms), whereas the stimulus of the other task was displayed until the participant switched to that other task. Response keys were clearly assigned to one of the two tasks (i.e., univalent responses were required)

In contrast to the common task switching paradigms (Kiesel et al., 2010), TSWP provides participants additionally with a preview to the task stimulus they have to respond to after the next task switch. That is, the stimulus of the task that will become relevant after the next task switch is already visible, while participants still work on the currently relevant task. A scheme of an exemplary stimulus presentation is illustrated in Fig. 2 (left). As becomes evident from this figure, each block of the TSWP starts with the concurrent presentation of one stimulus of each task, A and B, the participant will have to perform, for example, a letter and a digit classification task, as shown in Fig. 2. The participant is required to work on the two tasks in a predictable AAABBB sequence, corresponding to an alternating runs scheme in classic task-switching research (Rogers & Monsell, 1995). To guide the participants through this sequence, an additional arrow cue is provided, which marks the task that needs to be performed in the current trial (see Fig. 2). Responses to the two tasks (yes, no) have to be provided with the index and middle fingers of different hands (e.g., left hand for task A and right hand for task B). Upon each given response to the marked task, the stimulus of this task is immediately updated in the subsequent trial, that is, without any response–stimulus interval. In the meantime, the stimulus of the other task remains visible throughout all three trials of the currently marked task. After the third trial, a shift of the arrow to the other task indicates that the participant finally has to switch to exactly this other task stimulus, and to start the work on the three trials of this task. Thus, the remaining stimulus of the respective other task always provides a “preview” of what the specific task will be after the next switch. Participants are instructed to work as quickly and accurately as possible through the sequence of the two tasks for a predetermined time. However, no instructions are provided whatsoever about the use of the information provided by the preview stimulus. Thus, the participants are completely free with respect to process or to ignore this information. Accordingly, it was up to them either to use the preview stimulus to prepare the switch while still working on the currently relevant task, or to ignore that stimulus and process it after the switch. A demonstration of the paradigm^1^ is provided on the open science framework platform: https://osf.io/sb6wq/.

#### Free concurrent dual-tasking (FCDT)

The general features of the presentation of task stimuli and the response recording correspond to the one in the TSWP paradigm. However, in contrast to the TSWP, the participants are not requested to work on both tasks in a predefined sequence, but are also completely free in respect to when and how long they want to work on a given task. They are only instructed to maximize their throughput of both tasks within the given time without neglecting one task in favor of the other, but do not get any instructions whatsoever how to organize the coordination of tasks in order to achieve this goal. Thus, the participants can freely organize their responses to the two tasks with the only constraint that they should perform the two tasks with the same priority. A typical scheme of stimulus presentation is presented in Fig. 2 (right). Like in the TSWP, each FCDT block starts with the presentation of one stimulus of each task. However, no additional cue is provided to mark the currently relevant task. Instead, it is up to the participants to decide which task to start with and after how many trials to switch between tasks. Upon each response to one of the tasks, only the task stimulus of the task just responded to is updated, whereas the task stimulus of the other task remains visible until the participants finally decide to switch to that task. Given that the responses to the two tasks are provided by different hands, the specific sequence of responses to the two tasks can then be derived post hoc from the timeline of response recordings. This, in turn, provides the basis for the identification of different sorts of response organization in terms of blocking, switching, or response grouping. Note that, like in the TSWP paradigm, the participants always have the opportunity for overlapping processing of the stimuli of both tasks, which might be used or not to optimize task-switching performance. Thus, the paradigm is suitable for analyzing the extent to which the preferred mode of cognitive task processing, as identified in the more controlled TSWP paradigm, also transfers to a situation of free dual-tasking and how this mode relates to the pattern of self-organized response scheduling. A demonstration of the paradigm is provided on the open science framework platform: https://osf.io/e6wgx/.

### Tasks

The experiment contained two simple classification tasks, which were used in both, the TSWP and the FCDT paradigm. The digit classification task consisted of a set of digits, which had to be classified according to their parity (2, 4, 6, or 8 vs. 3, 5, 7, or 9). In the letter classification task, participants had to decide whether a presented letter was a vowel or a consonant (A, E, I, U vs. G, K, M, or R).

### Stimuli and apparatus

The experimental stimuli were displayed in light grey (RGB = 245, 245, 245; font size = 24 px) on dark grey background (RGB = 90, 90, 90) on an Acer LCD screen (1280 × 1024 px, sampling with 60 Hz). In single-task blocks, the stimuli were presented in the center of the screen. All dual-task^2^ blocks started with the simultaneous presentation of stimuli for both tasks. The stimuli were then presented vertically with close spatial proximity (distance = 16 px), allowing concurrent perception of the two stimuli without eye movements. Stimulus presentation and response recording were controlled by a custom-made JAVA software running on an Intel Pentium (2.9 GHz, 8 GB RAM; Windows 7 Pro). Participants responded by pressing predefined letter keys on a standard keyboard. The keys ‘K’ and ‘L’ were used with the index and middle finger of the right hand to respond to one task and the keys ‘S’ and ‘A’ with the index and middle finger of the left hand to respond to the other task. The task-hand assignment was counterbalanced between participants. The keys were marked with color points for easier recognition.

### Procedure

One to three participants were tested simultaneously at independent PC workstations, separated by opaque screens. The experiment was structured into three parts: (1) introduction and practice of the single tasks, (2) introduction and practice of the FCDT paradigm followed by the experimental phase, and (3) introduction and practice of the TSWP paradigm followed by the experimental phase. Note that all participants received the same order of the two paradigms to avoid that their voluntarily exhibited response sequence pattern in the FCDT would be influenced by the prescribed response sequence in the TSWP paradigm.

Throughout training and experimental phase, the single-task and dual-task trials had to be performed for a specified time rather than a specified number of trials. Performance assessments were based on task throughput, which is the number of trials correctly performed within the given time, reflecting both speed as well as accuracy. Accordingly, participants were instructed to maximize their performance in terms of number of correct responses to one or both tasks within the given time.

Instructions for the single tasks and the two paradigms were presented on the computer screen and could be read self-paced. The training of both single tasks comprised a 30-s block for task familiarization and a subsequent 60-s block for further practice, respectively. The subsequent training and experimental phase of both paradigms followed the same general block structure. For both paradigms, the initial practice phase included a 60-s dual-task block for task familiarization and an additional 120-s dual-task block for further practice, respectively. Following this practice phase, the experimental phase for each paradigm included three runs. Every run comprised two 120-s dual-task blocks followed by one 60-s block of each single task. All runs began with the dual-task condition to prevent the influence of practice effects in the two single tasks biasing the identification of processing modes. The single-task blocks were included to control for stability of single-task performance. The order of single tasks was counterbalanced across these blocks.

The task stimuli of each block were randomly drawn from the stimulus sets of the respective tasks with the constraint that no stimulus would be directly repeated and that the two possible responses per task were equally distributed. During single- and dual-task blocks, task stimuli were shown until a response was recorded. Upon a recorded response, the next stimulus appeared immediately (response–stimulus interval = 0 ms).

After every block, participants were provided with feedback on the number of performed trials and the number and the percentage of correct responses of each task for 5 s. Feedback was provided primarily to maintain the participants’ motivation and to inform them about their error rates to ensure that participants not only speed up their responses, but also try to keep their error rates within reasonable limits. Throughout the experimental procedure, short breaks of 1 and 2 min were included between the experimental runs and between the two paradigms, respectively. Altogether, the experiment lasted approximately one and a half hour.

### Design

All participants performed both paradigms. Participants were then post hoc categorized into subgroups, separately for each dual-task paradigm. Based on their performance in the TSWP, we classified participants regarding their mode of task processing depending on the degree to which they used the preview (i.e., serial, semi-overlapping, or overlapping). In the FCDT, we classified participants regarding their strategy of response organization based on their response pattern (i.e., blocking, switching, or response groupers). The resulting 3 (categorization in FCDT) × 3 (categorization in TSWP) contingency table then was used for analysis of the proposed correspondence between both levels of task coordination.

### Data analyses

For both paradigms, three different trial types were considered: single-task trials, repetition trials, and switch trials. Repetition trials included all trials in which participants performed the same task as on the previous trial. Switch trials were defined as trials in which participants performed a different task compared to the previous trial. For each single-task block and the different trial types in dual-task blocks, the mean inter-response intervals (IRIs), defined as the time interval between two subsequent responses, and error rates (ERs), defined as the number of incorrect responses compared to the total number of responses given, were calculated for each participant. IRIs had to be used instead of reaction times that are usually calculated as the interval between stimulus and response. This was necessary, because in the FCDT paradigm, one or more responses to the other task could occur between the onset of the preview of the switch stimulus and the response to this switch stimulus. Hence, IRIs were more appropriate to assess the time needed for responding to a task. Only correct responses were considered in the analyses of both, IRIs, and measures of efficiency. In both paradigms, the data of the different trial types of each participant were aggregated across tasks and experimental runs. Averaged across tasks and participants, this yielded 266 single-task trials (SD 31) and 488 dual-task trials (SD 84.6) for the TSWP paradigm. For the FCDT paradigm, 254 single-task trials (SD 30) and 473 dual-task trials (SD 79.5) averaged across tasks and participants were available. Regarding the identification of outliers at single trial level, first, all trials with an IRI longer than 5 s were discarded. Subsequently, trials slower than two SD from the participant’s mean IRI in the according trial type were excluded. This yielded in excluding 4.5% of trials (SD 0.8%) in the FCDT and 4.4% of trials (SD 0.7%) in the TSWP paradigm per participant on average.

#### Identification of individually preferred modes of task processing in the TSWP

The identification of individually preferred modes of task processing was based on a fine-grained analysis of the overtly observable response time patterns, following the rational and criteria described in our previous study (Brüning & Manzey, 2018). In a first step, the switch-trial data were inspected for specific cues, the so-called fast switches that provide a first indicator of possible overlapping processing. For this purpose, we compared each switch-trial IRI with the first quartile of the distribution of IRIs in the respective single-task block of a participant. Only switch trials with an IRI at least as fast as the 25% quickest responses in the respective single-task trials were classified as fast switches. Since such fast switches are considerably shorter than the mean processing time needed for a single-task response, it can be assumed that not only task-set reconfiguration and/or overcoming possible task-inertia effects from the preceding task (Allport, Styles, & Hsieh, 1994; Monsell, 2003), but also some stimulus processing must have been carried out prior to that fast switch IRI. Based on the outlined analysis, the fast switch rate of an individual was calculated by relating the number of fast switches to the number of all switches performed by the individual.

**Fig. 3:**
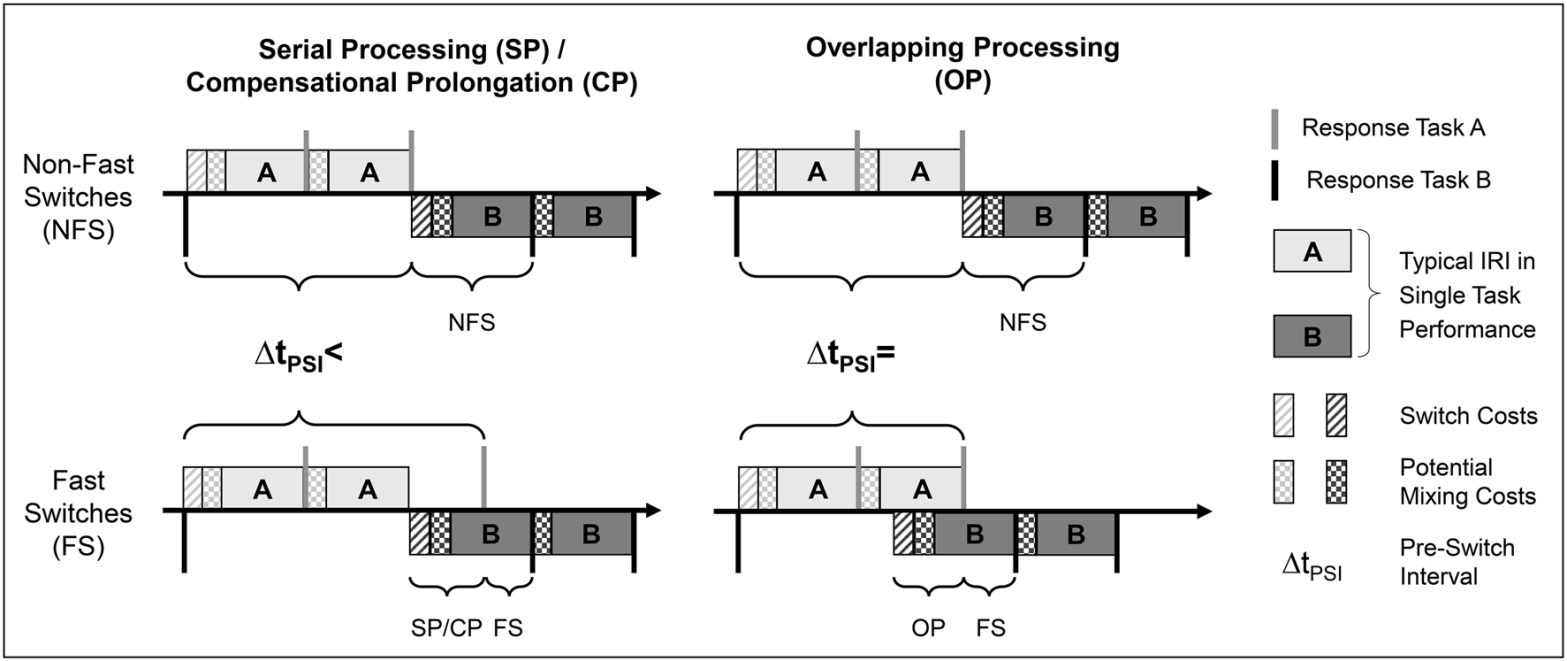
Scheme of the comparisons performed to distinguish serial processing and potential compensational prolongations (timelines on the left side) from actual overlapping processing (timelines on the right side). The schematic timelines include two responses to task A, followed by a switch and two responses to task B. The comparisons are made between the pre-switch intervals (PSI) preceding non-fast switches (upper row) and fast switches (lower row), respectively. Note that, in case of overlapping processing, the specific processing steps assumed to take place in an overlapping manner (patterned squares) are shown directly before the switch for illustrative purposes. We do not hold specific assumptions about when the stimulus preview will be used

However, before considering the fast switch rate as indicator of the degree of overlapping processing for a single participant, two alternative sources of fast switches, a compensational prolongation in trials directly preceding the switch (i.e., in the pre-switch interval) or a production just by chance (e.g., due to unrelated muscular pre-activation), had to be excluded. To rule out the former, we tested whether participants showed longer mean responses in the interval before a fast switch than before all non-fast switches. The underlying logic is that the pre-switch interval (PSI) preceding a non-fast switch could entail mixing costs resulting from the performance of two tasks rather than one, but should not lead to other time losses. In contrast, the PSI preceding a fast switch could include a combination of mixing costs and potential compensational prolongations in case of interleaving, but still serial processing. Only if no compensational prolongation can be found in the PSI of a fast switch compared to non-fast switches, overlapping processing must have been applied. Accordingly, the comparison of PSIs preceding fast and non-fast switches allows for identifying such compensational prolongations without confounding them with potential mixing costs. The comparisons are schematically depicted in Fig. 3. Finally, only those participants whose mean PSI of fast switches was equal or shorter than their according mean PSI of all non-fast switches, were considered to be overlapping processors.

The second alternative, that is, that fast switches might have occurred randomly, was addressed by comparing the individual’s fast switch rate found in the present TSWP paradigm with a distribution of fast switch rates produced by chance. This latter distribution was obtained from a sample performing task switching without a preview option, thus only allowing for serial processing. In the present study, we used data acquired in our previous study (Brüning & Manzey, 2018), which included a sample of *N* = 46 participants performing an alternating runs task-switching paradigm using similar simple classification tasks as in the current study but without providing a preview. Without the option to use a preview, the participants of this control group were forced to work serially on the two tasks. Only those individuals of the current study whose fast switch rate was three SDs above the grand mean of the random distribution derived from the control group (14.61%) were finally classified as overlapping processors. In contrast, individuals showing a fast switch rate in the present TSWP paradigm, which fitted to the distribution of incidental fast switches by being lower than the grand mean plus one SD (6.55%) were still defined as serial processors. All remaining participants were considered to show too many fast switches to occur by chance, but too few to indicate a manifest and clear preference for an overlapping processing mode, and, thus were classified as semi-overlapping processors. Note that our criteria, combined with the procedural aspect that single-task blocks always followed dual-task blocks, altogether represent a relatively conservative indicator of overlapping processing.

#### Identification of individually preferred strategies of response organization in the FCDT

The identification of preferred strategies of response organization was based on post hoc analyses of the response sequences over time produced by the individual participants in the FCDT. This included how often participants repeated a given task and how they organized task switches. Capitalizing on the approach and criteria introduced by Reissland and Manzey (2016), we inspected the switch rates of participants to distinguish between blockers and switchers, but also considered the distribution of IRIs in switch trials to distinguish response groupers from switchers, as well. The switch rate was defined as the number of switches related to the maximum number of switches that would have been possible given the number of each task performed in the given time. Participants who showed a switch rate below 10% and, thus, minimized the number of task switches while multitasking, were classified as blockers as they obviously preferred to separate the performance of both tasks as much as possible. In contrast, all individuals with a switch rate higher than 10% and an unimodal distribution of IRIs in switch trials were classified as switchers. In this case, most of the switch IRIs fluctuate evenly around the mean IRI of switch trials. Finally, individuals who performed a high number of switches (i.e., switch rate > 50%) along with a bimodal distribution of switch IRIs were classified as response groupers. They typically produced prolonged switch IRIs while processing the stimuli of both tasks internally, followed by a very short response when they finally have processed both tasks and executed the according response in close succession. To distinguish response groupers from switchers, we tested whether the distribution of switch IRIs deviated from a unimodal distribution (i.e., most likely bimodal) by means of Hartigan’s dip test (Hartigan & Hartigan, 1985). Since such tests are highly sensitive for signs that contradict unimodal distributions, we considered a *p* value of *p* < 0.001 as critical to confirm bimodality. However, all tests for bimodality were also confirmed by visual inspection. For an illustration of the strategies, compare the stereotype sequences of responses to both tasks as shown in Fig. 1 in Sect. 1.

#### Analyses of multitasking efficiency

The multitasking efficiency achieved by individual participants in TSWP and FCDT was assessed by the overall dual-task performance efficiency (ODTPE) measure proposed by Reissland and Manzey (2016) and refined by Brüning and Manzey (2018). It describes how many trials of the digit and letter classification tasks which a participant can perform correctly in the 2-min dual-task blocks (TSWP or FCDT), relative to the overall number of correct trials achieved in the two 1-min single-task blocks. Thereby, it represents a straightforward throughput measure, considering speed and accuracy of responses equally. Positive ODTPE scores indicate that the task throughput in dual-task blocks is higher than in single-task blocks, thus reflecting multitasking benefits. Negative ODTPE scores indicate multitasking costs, and ODTPE scores = 0 indicate that the task throughput in dual-task blocks is the same as in single-task blocks. A detailed description of this measure can be found in the appendix of our previous study (Brüning & Manzey, 2018).

## Results

### Correspondence between modes of processing and response organization strategies

The categorization procedure for individually preferred modes of processing in TSWP resulted in 31 serial processors, 13 semi-overlapping processors, and 26 overlapping processors. In the FCDT paradigm, the categorization of the individuals regarding their preferred strategy of response organization revealed that 32 individuals preferred a blocking strategy, 22 individuals preferred a switching strategy, and 16 participants chose a response grouping strategy. Overall, the relative size of the subgroups resembled those found in previous studies (Brüning & Manzey, 2018; Reissland & Manzey, 2016). Table 1 shows the contingency between processing modes and response organization strategies.

**Table 1:** Number of participants per subgroup in the TSWP and the FCDT paradigm

	Processors	FCDT (behavioral)			Total
		Blocker	Response grouper	Switcher	
TSWP (cognitive)	Serial	23	4	4	31
	Semi-overlapping	7	2	4	13
	Overlapping	2	10	14	26
Total		32	16	22	70

A Chi-square test revealed a significant contingency between the distribution of the used processing modes and response organization strategies (*χ*^2^ (4, *N* = 70) = 25.89, *p* < 0.001). Almost three-quarters of serial processors indeed preferred a blocking response organization strategy (*n* = 23 out of 31, i.e., 74%), instead of one of the interleaving strategies (switching or response grouping; 26%) strategy. Comparably, the vast majority of the overlapping processors chose a switching or response grouping strategy (92%), whereas only a minority of two individuals (8%) preferred blocking. By contrast, the semi-overlapping processors, for whom we did not hold specific expectations, did not show a clear preference for either interleaving or blocking response organization strategies.

### Effects of individual preferences on multitasking efficiency

**Fig. 4:**
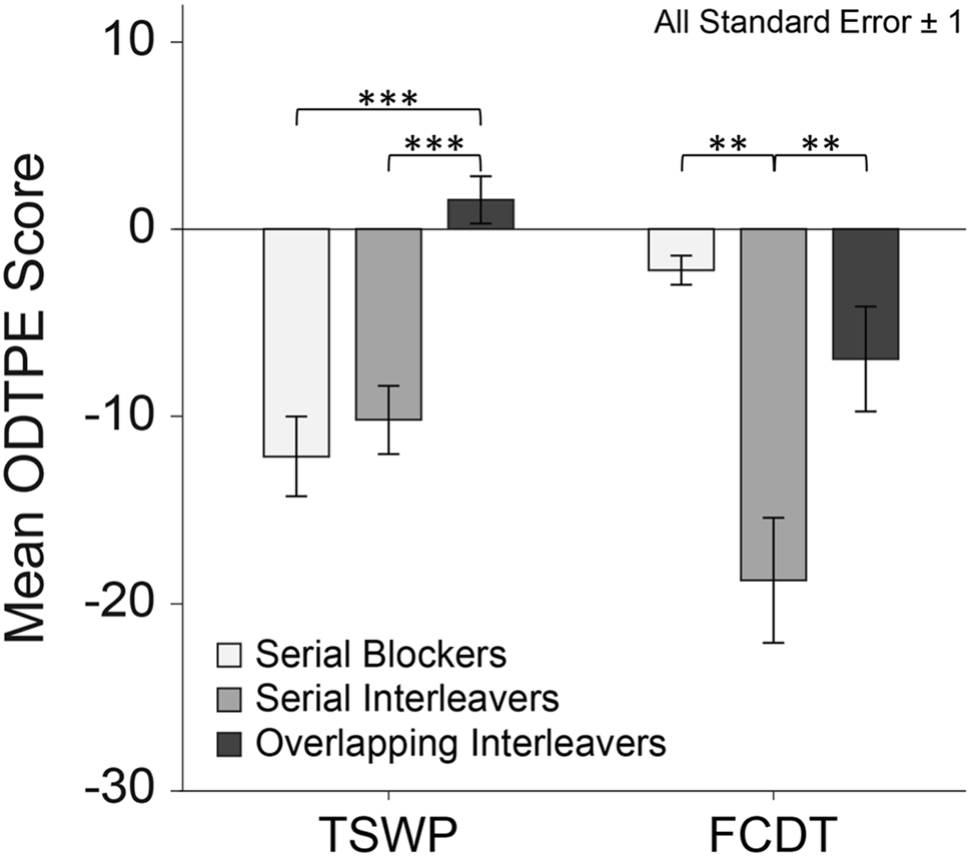
Mean overall dual-task performance efficiency (ODTPE) scores achieved by the serial blockers, serial interleavers, and overlapping interleavers in the task-switching with preview (TSWP) paradigm and the free concurrent dual-tasking (FCDT) paradigm. Error bars represent ± one standard error. *p < 0.05. **p < 0.01. ***p < 0.001

A second set of analyses addressed the impact of the preferred mode of task processing combined with a preferred strategy of response organization on the multitasking efficiency in the different paradigms. For this purpose, the mean ODTPE scores achieved in the two paradigms by the three largest subgroups, which are, serial blockers (*n* = 23), serial interleavers (8), and overlapping interleavers (24), were inspected (Fig. 4) and analyzed by a mixed 3 (subgroup) × 2 (paradigm) ANOVA, with the second factor defined as a within-subjects factor.

The subgroups varied considerably in their size, and both the Levene test, *F*(2, 52) = 6.36, *p* = 0.003, and the Box test, *F*(6, 3797.66) = 3.46, *p* = 0.002, were significant. Therefore, a robust implementation of a heteroscedastic mixed ANOVA based on trimmed means (*M*_*t*_) suggested by Mair and Wilcox (2019) was used, based on the R package WRS2. This ANOVA revealed a significant main effect of subgroups, *F*(2, 28.50) = 14.88, *p* < 0.001, as well as a significant Subgroup x Paradigm interaction, *F*(2, 27.82) = 21.34, *p* < 0.001. The main effect of paradigm was not significant, *F*(1, 38.84) = 2.49, *p* = 0.123.

Subsequent robust one-way ANOVAs with the subgroups as between-subject factor were performed separately for the two paradigms to further explore the basis of the interaction effect. As described in more detail in the method section, reported means of ODTPE scores represent the relation of achieved number of correct responses in dual-task blocks compared to single-task performance (e.g., positive values reflecting higher throughput during multitasking). The one-way ANOVA calculated for the ODTPE scores obtained from TSWP revealed that the subgroups differed significantly, *F*(2, 16.70) = 27.62, *p* < 0.001, *ε*^2^ = 0.79, with considerably better multitasking efficiency shown by the overlapping interleavers (*M*_*t*_ = 1.6%, standard error, SE 1.3), than by the serial interleavers (*M*_*t*_ = − 10.2%, SE 1.8) and the serial blockers (*M*_*t*_ = − 12.1%, SE 2.1). Post hoc pairwise comparisons based on the Fisher’s Least Significant Difference Test (LSD; Howell, 2016) on the trimmed means confirmed that overlapping interleavers differed significantly from serial blockers and serial interleavers (both *p* < 0.001), whereas the two latter groups did not differ from each other (*p* = 0.441).

The one-way ANOVA calculated for the ODTPE scores obtained from FCDT again revealed a significant main effect of subgroups, *F*(2, 11.33) = 17.00, *p* < 0.001, *ε*^2^ = 0.68, yet with another pattern of differences than in the analysis of the TSWP data. This time, the best multitasking efficiency was achieved by the serial blockers (*M*_*t*_ = − 2.2%, SE 0.8), followed by the overlapping interleavers (*M*_*t*_ = − 7.0%, SE 2.8) and the serial interleavers (*M*_*t*_ = − 18.8%, SE 3.3). Post hoc pairwise comparisons with LSD correction of the trimmed means between the efficiency of serial blockers and overlapping interleavers failed to reach the conventional level of significance (*p *= 0.091). However, serial interleavers showed a significantly worse multitasking efficiency, compared to both other groups (both *p* < 0.01). While it was expected that serial interleavers would perform worst in self-organized multitasking, the finding that overlapping interleavers could not outperform the serial blockers was surprising. Obviously, overlapping interleavers were not able to transfer their advantages with respect to multitasking efficiency achieved by overlapping processing in the TSWP paradigm to a situation demanding self-organized response scheduling in addition.

In order to better understand the performance difference between the two paradigms, we conducted a further analysis. For this purpose, the mean IRIs and ERs for single-task and dual-task trials and the mean fast switch rate of the overlapping interleavers in the TSWP and in the FCDT paradigm were contrasted. Individuals applying a response grouping strategy in the FCDT as well as three switchers had to be excluded from this analysis, because they did not produce enough repetition trials for a direct comparison of performance between the two paradigms. The remaining 11 individuals of this subgroup who showed a preference for overlapping processing in the TSWP combined with a switching strategy involving a considerable number of repetitions in the FCDT were considered in this analysis. The results of this analysis are shown in Table 2.

**Table 2:** For 11 overlapping switchers, means of inter-response intervals (IRI) and error rates (ER) in single-task and dual-task trial types (repetition and switch), as well as their mean rate of fast switches in TSWP and FCDT are shown

Trial type	TSWP		FCDT	
	IRI	ER	IRI	ER
Single task	584	3.2	611	2.6
Repetition	573	2.0	657	2.5
Switch	612	2.7	627	2.7
% Fast switches	34.5		45.2	

As becomes evident, the already high percentage of fast switches (*M* = 34.5%, SE 4.8) in the TSWP paradigm, was even more pronounced in the FCDT paradigm (*M* = 45.2%, SE 4.8). This led to small multitasking costs in terms of mean time losses when switching between the tasks compared to the single-task performance in both paradigms (TSWP: *M* = 28 ms, SE 16; FCDT: *M* = 16 ms, SE 34). A paired *t* test comparing the mean time losses in switch trials between both paradigms was not significant, *t*(10) = 0.33, *p* = 0.749, *d* = 0.1.

In contrast, the repetition trials performed by overlapping switchers in FCDT were on average 46 ms longer (SE 14) compared to single-task trials. This marked a sharp contrast to the TSWP paradigm, where no such costs emerged and where the mean IRI for these trials were even 10 ms (SE 5) shorter than the according single-task responses. A paired *t* test comparing these effects across the two paradigms was significant, *t*(10) = 4.49, *p* = 0.001, *d* = 1.35. One explanation of the prolongation observed in the repetition trials in the FCDT might be that individuals performing overlapping processing in the TSWP were not able to do so in the FCDT. However, this explanation was ruled out by the finding that the increased costs associated with task repetitions in the intervals before the switch emerged independent of the time needed for a switch (mean repetition costs before fast switches: *M* = 50 ms; SE 16 vs. before all other switches: *M* = 49 ms, SE 15; *t*(10) = − 0.14, *p* = 0.894, *d* = 0.04). Moreover, no comparable effect was found by comparing the mean times for repetition trials of the serial blockers in the TSWP and FCDT paradigm. Participants of this latter group worked even slightly faster on repetition trials than on single-task trials in both paradigms (TSWP: *M* = 607 ms; SE 15 vs. *M* = 631 ms; SE 14; FCDT: *M* = 667 ms; SE 17 vs., *M* = 672 ms; SE 17). Overall, these findings suggest that the decreased multitasking efficiency of overlapping switchers in the FCDT compared to the TSWP paradigm was not due to differences at switch trials but due to new costs arising in the repetition trials in this group.

## Discussion

The present study was guided by two objectives. First, the experiment was conducted to investigate the hypothesis that preferred modes of task processing and strategies of response organization were systematically linked; that is, they would reflect interwoven aspects of individual differences in multitasking. As will be discussed in some detail below, the results support this hypothesis to a large extent. The second objective was to investigate the hypothesis that the specific combination of preferred mode of task processing and strategy of response organization would determine the multitasking efficiency. This hypothesis was only partially supported by the results.

To investigate the first objective, the preferences of each individual participant for both aspects of task coordination were probed based on two different paradigms. The two paradigms reflected different degrees of freedom for the participants to self-organize their performance of two tasks. The TSWP only demanded task coordination at the level of task processing, whereas the FCDT put additional demands on the level of response organization. The identification of the task processing mode and the response organization strategy individuals preferred followed the rationales described by Reissland and Manzey (2016) and Brüning and Manzey (2018), enriched by the introduction of two additional statistical criteria (i.e., the PSI and the use of Hartigan’s Dip Test) to further objectify the procedure. The results directly confirm the findings of our previous research. Two subgroups differing in their spontaneous preference for serial versus overlapping processing could be identified based on the use of preview in the TSWP, and again, three different types of strategies of response organization (blocker, switchers, and response groupers) emerged in the FCDT paradigm.

Even more important, the data also supported our assumption that individuals applying serial versus overlapping processing in the TSWP paradigm differed in their preferred strategy for organizing the responses to the same two tasks in the FCDT paradigm. As expected, the vast majority (74%) of participants who processed the tasks in the TSWP in a strict serial manner showed a strong preference for blocking their responses to the two tasks in the FCDT. This allowed them to keep the two tasks as separate as possible even in the multitasking situation. In contrast, and also in accordance with our expectations, 24 out of 26 individuals using the preview option for overlapping processing in the TSWP preferred a sort of task-interleaving strategy, that is, either switching or response grouping, in the FCDT. Both effects provide evidence for our general hypothesis that humans tend to organize their responses to two concurrent tasks in a way that is supported by their preferred mode of task processing. However, there are also a noteworthy, albeit small number of serial and overlapping processors in TSWP who have chosen a response strategy under conditions of self-organized multitasking that was not in accordance with our predictions. This in turn emphasizes the fact that the preferences for the two aspects of task coordination, task processing and response organization, are clearly interwoven, but do not simply represent interchangeable aspects of individual differences in multitasking.

The second aim of the current study was to investigate how different combinations of individually preferred modes of task processing and strategies of response organization would affect the multitasking efficiency of participants. The multitasking efficiency was assessed by comparing the task throughput (i.e., number of correct responses in the given time) achieved by a participant while multitasking compared to single-task performance. Based on the results of our previous research (Brüning & Manzey, 2018; Reissland & Manzey, 2016), we expected overlapping processors to perform more efficiently than serial processors in the TSWP paradigm, because they can realize time gains particularly at task switches. Consequently, we also expected overlapping processors to achieve multitasking benefits in the FCDT when choosing a strategy of interleaving both tasks by either switching frequently between both tasks or by applying response grouping. Both strategies might benefit from overlapping processing. In contrast, serial processors in the TSWP were expected to prefer a blocking strategy of response organization in self-organized multitasking to reduce the number of switches between the tasks and thus minimize time losses due to task switches. As a result of these disadvantages of serial processing when using interleaving strategies of response organization, serial processors were expected to perform worst of all subgroups when choosing one of the task-interleaving strategies.

Our results, however, only partially support these assumptions. As expected, applying overlapping processing using the preview in the TSWP paradigm led to significantly better multitasking performance than serial processing. Furthermore, our data also confirm the assumption that serial processors as identified in the TSWP showed worst multitasking performance in terms of highest multitasking costs when applying a switching or response grouping strategy in the FCDT. This finding is most plausible, since the combination of serial processing with a switching or response grouping strategy involves substantial time losses when switching between task, which in turn accumulate over a high number of switch trials. Accordingly, the fact that serial interleavers perform poorly in terms of multitasking efficiency is not surprising. The fact that some serial processors used an interleaving strategy of response organization, at all, suggests that they had not been aware of their relatively inefficient performance. Indeed, research on metacognition of multitasking in controlled laboratory settings (Finley, Benjamin, & McCarley, 2014) as well as in the field (Horrey, Lesch, & Garabet, 2008, 2009; Lesch & Hancock, 2004) shows that people might not necessarily have metacognitive insight on the extent to which they are personally vulnerable to the risks of multitasking. Similarly, also in the PRP paradigm, characterized by high control of timing of tasks, participants often are apparently not aware of their multitasking costs in dual-task settings (Bryce & Bratzke, 2014, 2017; Corallo, Sackur, Dehaene, & Sigman, 2008). This might also explain why, in the debriefing of the experiment, not only most of the serial blockers and overlapping interleavers, but also most of the serial interleavers reported to have chosen this particular response strategy to perform more efficient. However, since the participants were not requested to predict or assess their own performance efficiency in the course of the present study, further studies are necessary to determine whether individuals differing in their preferences for task coordination differ in their accuracy of introspective knowledge, as well.

In clear contrast to our expectations, the combination of overlapping processing and interleaving response strategies was not more beneficial than the combination of serial processing and the blocking strategy. At least descriptively, the subgroup of serial blockers even showed the highest performance efficiency of the three largest subgroups in the FCDT. They performed the two tasks with about the same task throughput as in the single-task condition. Based on their preference for a serial mode of task processing, we expected that they would not be able to realize any multitasking benefits, but would be prone to considerable cost effects when switching between tasks in self-organized multitasking. However, using a blocking strategy in the FCDT, serial blockers reduced the number of task switches to a minimum to cope with the multitasking demand. As a result, they minimized the risk of time costs when switching, so that overall neither the benefits nor the costs of multitasking arose.

Nonetheless, the overlapping interleavers did not outperform the serial blockers in the FCDT. This effect emerged, although the overlapping processors significantly outperformed the serial processors in the TSWP paradigm and although they applied a similar strategy of using preview and realizing a considerable number of fast switches in the FCDT as in the TSWP paradigm. Indeed, our additional analysis of the time gains and losses of overlapping switchers in the TSWP and FCDT paradigms revealed that their multitasking costs were obviously not caused by time losses at task switches, but by time losses in repetition trials. Furthermore, these latter repetitions costs emerged independent of the length of the next switch response, which rules out that the overlapping processors were less able of overlapping processing in the FCDT than the TSWP paradigm. Therefore, what might have been the cause of these cost effects? One might argue that these repetition costs reflect typical mixing costs, which sometimes have been reported from task-switching studies resulting from the demand to keep two task-sets active in memory (Kiesel et al., 2010). However, the fact that both subgroups did not show similar costs in the TSWP renders this interpretation not very plausible. Furthermore, such costs did not emerge in the FCDT data of our serial blockers, despite the fact that they also were requested to maintain the task-sets of both tasks active in memory. Therefore, the cost effects observed in repetition trials of overlapping switchers must be related to multitasking demands in the FCDT that are specific for the switching strategy.

The most obvious difference between the switching and the blocking strategy in this respect is that switchers had to organize their responses and the scheduling of switches “online” while performing the two tasks concurrently. For this purpose, they must have monitored their performance and have chosen their exact order of responses throughout a dual-task block. In contrast, the blocking strategy can be planned beforehand with only minimal demands on online monitoring. We suppose that the repetition costs visible in the data of the overlapping switchers reflect the additional demands involved in monitoring and decision-making about switches associated with a switching strategy. This would also be in line with results from the voluntary task switching paradigm, suggesting that already the act of choosing the task to be performed incorporates top-down processes that generate time losses separable from switch costs (Arrington & Logan, 2004, 2005). Similar costs can also be found in PRP research showing that the coordination of the processing order of two tasks requires additional control processes (Kübler et al., 2018; Szameitat, Lepsien, Cramon, Sterr, & Schubert, 2006; Szameitat, Schubert, Müller, & Cramon, 2002). In sum, these additional demands on the cognitive control might explain the sustained cost effects found for overlapping switchers under conditions of self-organized multitasking. However, as cognitive control demands were not directly manipulated and investigated in the current study, the data are not conclusive in this regard. Nonetheless, if costs due to self-organization could be overcome, for example, through training, overlapping processing might eventually develop its potential and increase overall multitasking efficiency.

## Conclusions

To conclude, the results of the present study again confirm earlier findings of individual differences in multitasking, related to how cognitive processes are organized (serially vs. overlapping) and how responses to the tasks are scheduled (separating vs. interleaving). As a new insight, they also provide strong evidence for a correspondence between the individual preferences for these two aspects of task coordination. The majority of individuals preferring a serial processing mode in the more controlled task-switching with preview paradigm preferred a blocking strategy in the free concurrent dual-tasking paradigm, successfully reducing the dual-task demands. Comparably, most individuals preferring overlapping processing preferred an interleaving response organization strategy. This obvious link suggests that individual preferences emerging in both aspects of task coordination might reflect a common basic difference in the individually preferred style of cognitive control, corresponding to the distinction of flexibility and persistence as different styles of cognitive control proposed by Hommel (2015). The sources of these differences are difficult to derive from the currently available data and, thus, are a matter of speculation at the present time. As Hommel (2015) suggests, they might include environmental influences like cultural differences and/or learning experiences, or even genetic predispositions. However, overlapping processors were not consistently able to transfer their performance advantages in terms of time gains at switches to a self-organized context due to the choice of a non-optimal response scheduling and/or new costs. The latter probably resulted from increased performance monitoring demands in completely self-organized multitasking. Further research should try to identify relevant context variables affecting the relative benefits and costs of different sorts of task coordination in terms of multitasking efficiency.

Another interesting question for further research regards possible links of the preferences found at the different levels of task coordination to other cognitive abilities (e.g., working memory, processing speed, or fluid intelligence) or even personality factors (e.g., resistance to change, polychronicity, or motivational tendencies). Thus far, the role of cognitive abilities, personality characteristics, and different attitudes as possible predictors of multitasking performance has only been investigated with respect to comparatively complex work simulations (e.g., Hambrick, Oswald, Darowski, Rench, & Brou, 2010; König, Bühner, & Mürling, 2005; Mäntylä, 2013; Redick, 2016; Zimmermann, Kubik, Persson, & Mäntylä, 2019). The results suggest that particularly working memory capacity can predict the performance in complex multitasks. However, the performance indicators considered in these studies were usually limited to global performance scores, which do not provide much insight in how the superior multitasking performance is achieved with higher working memory capacity. Identifying links between cognitive abilities, personality characteristics, preferred modes of task processing and strategies of response organization would nicely complement this line of research. For example, first hints were found that the preference for serial versus overlapping processing might be related to working memory capacity (Brüning & Manzey, 2018). In this sense, the emergence of different preferences for specific modes of processing and strategies of response organization would reflect mediating factors explaining how higher working memory capacity can be used to achieve better multitasking performance.

## Footnotes

1 The demonstration of the paradigms was created according to the procedure proposed by Heycke and Spitzer (2019).

2 We use the term “dual-task” to refer to all conditions where the participants had to perform both tasks, either concurrently in a self-organized manner (FCDT), or according to the given AAABBB schedule (TSWP).
